# Iron Matters: Comparative Impact of Beta-Adrenergic Stimulation and Iron Chelation on Cardiac Iron Metabolism and Mitochondrial Function

**DOI:** 10.3390/biom16040582

**Published:** 2026-04-14

**Authors:** Josep Francesch-Manzano, Marta Tajes, Raúl Ramos-Polo, Cristina Enjuanes, Maria del Mar Ras-Jiménez, Andreea Eunice Cosa, Katrin Marinova, Carla Enrich-Soria, Pedro Moliner, Laia Lorenzo-Esteller, Núria José-Bazán, Josep Comín-Colet

**Affiliations:** 1 Bio-Heart Cardiovascular Diseases Research Group, Bellvitge Biomedical Research Institute (IDIBELL), L’Hospitalet de Llobregat, 08908 Barcelona, Spain; 2 Department of Clinical Sciences, School of Medicine, Universitat de Barcelona, 08007 Barcelona, Spain; 3 Centro de Investigación Biomédica en Red de Enfermedades Cardiovasculares (CIBERCV), Instituto Salud Carlos III, 28029 Madrid, Spain; 4 Cardiology Department, Heart Institute, Bellvitge University Hospital, L’Hospitalet de Llobregat, 08907 Barcelona, Spain; 5 Community Heart Failure Program, Cardiology Department, Bellvitge University Hospital, L’Hospitalet de Llobregat, 08907 Barcelona, Spain; 6 Cardio-Oncology Unit, Catalan Institute of Oncology, Bellvitge University Hospital, L’Hospitalet de Llobregat, 08908 Barcelona, Spain; 7 Internal Medicine Department, Santa Caterina Hospital, Salt, 17190 Girona, Spain; 8 Department of Biomedical Engineering, Yale University, New Haven, CT 06511, USA

**Keywords:** heart failure, iron deficiency, cardiac cell, mitochondrial function, isoproterenol, deferoxamine

## Abstract

Iron deficiency (ID) is frequent in patients with heart failure (HF) and is correlated with adverse outcomes, yet its involvement in HF pathophysiology is not fully understood. Hyperactivity of the sympathetic nervous system (SNS) is the central feature of HF. We aimed to compare the effects of isoproterenol (ISO), a β-adrenergic agonist (SNS stimulation), with those of the iron chelator deferoxamine (DEF), to evaluate how β-adrenergic stimulation influences cardiac iron. In this study, H9c2 cardiac cells were challenged with ISO, DEF or both and several parameters related to iron metabolism were analyzed. In all cases, the cells decreased their intracellular iron levels. ISO induced alterations in key cardiac iron metabolism molecules that were, in most cases, comparable to those elicited by DEF, emphasizing the direct impact of β-adrenergic stimuli on iron metabolism and mitochondrial dysfunction. Nevertheless, unlike DEF, ISO triggered a shift in mitochondrial energy metabolism. These findings suggest that β-adrenergic stimulation, as a major component of neurohormonal activation, may contribute to the development of ID in cardiac cells, highlighting the importance of iron homeostasis and the need to further investigate iron dysregulation in this context.

## 1. Introduction

Heart failure (HF) is a severe clinical syndrome and a significant burden for public health systems [1]. The current understanding of its pathophysiology relies on the neurohormonal hypothesis, which proposes that HF progresses as a result of the persistent harmful and maladaptive consequences of chronic neurohormonal activation affecting the heart and the broader cardiovascular system [2,3]. Hyperactivity of the sympathetic nervous system is the central feature of HF. This leads to an elevation in catecholamines levels, which stimulates β-adrenergic receptors, initiating an adaptive compensatory response that transiently normalizes cardiac output through ventricular wall thickening [4]. However, sustained β-adrenergic receptor stimulation perpetuates the cardiac remodeling process, promoting ventricular dilation and the development of cardiac fibrosis. β-adrenergic stimulation using β-agonists such as isoproterenol (ISO) has been routinely used to experimentally induce cardiac disorders in in vitro and in vivo models and to test the efficacy of therapeutic strategies in HF [5,6].

Inhibiting these neurohormonal systems has demonstrated a reduction in morbidity and mortality among patients with HF and reduced left ventricle ejection fraction (HFrEF) and serves as the cornerstone of modern pharmacological therapy [7,8]. Nevertheless, these otherwise effective therapies have not succeeded in achieving complete symptom remission or in fully restoring life expectancy in many patients. The incidence and mortality rates of HF remain high [9], with epidemiological surveys indicating that 50% of HF patients die within 5 years [10,11]. This has driven growing interest in novel therapeutic targets. Notably, ID—affecting up to 50% of HF patients [12,13]—is linked to increased mortality and hospitalization [13,14], reduced functional capacity, and poorer health-related quality of life [15] irrespective of anemia status [13,15,16]. Interestingly, current guidelines recommend routine screening for ID in patients with chronic HF and the consideration of intravenous iron supplementation in symptomatic patients with confirmed deficiency [17], since correction alleviates HF symptoms, reduces the risk of hospitalization and improves quality of life [18,19,20]. Several factors contribute to ID, such as systemic inflammation, diminished dietary intake, and blood loss, but data explaining the high prevalence are lacking [21]. While ID has traditionally been viewed as an extracardiac comorbidity that complicates the course of HF, emerging evidence suggests that it may directly influence the mechanisms underlying HF onset and progression [22,23,24,25]. Works from our group previously identified association between neurohormonal activation and systemic ID [22]. In patients with chronic HF, heightened sympathetic activity has been shown to correlate with ID independently of anemia. Moreover, other studies report a 20–30% reduction in myocardial iron content in individuals with advanced HF [26]. A primary reason for identifying ID in patients with HF is to correct intracellular iron depletion within cardiomyocytes or skeletal muscle, as iron is required for the synthesis of heme and iron–sulfur clusters that are essential for ATP production and the maintenance of contractile function [27]. Beyond its role in oxygen transport, iron is essential for cellular metabolism, particularly as a mitochondrial cofactor involved in oxidative stress regulation and ATP synthesis [24,28]. Reactive oxygen species (ROS) disrupt mitochondrial Ca^2+^ homeostasis, thereby impairing contractility and increasing myocardial stiffness [29]. Hence, iron homeostasis is critical for cells with high energy demand, such as cardiomyocytes [13,24].

Despite the extensive data accumulation, the contribution of iron in the pathophysiology of HF remains poorly characterized. Previously, our group showed an alteration of cardiac iron metabolism in experimental models of neurohormonal activation [30].

Here, we hypothesized that β-adrenergic stimulation, as a main component of neurohormonal activation, may contribute to the generation of ID in cardiac cells and mitochondrial impairment. Accordingly, this study aimed to compare the effects of β-adrenergic stimulation and an iron chelator in cardiac cells to assess the contribution of iron regulation to the cellular adaptations triggered by adrenergic signals.

## 2. Materials and Methods

### 2.1. Cell Culture Model

Rat embryonic heart-derived H9c2 cells (from ATCC-Massanas, VA, USA; CRL-1446) were cultured in Dulbecco’s modified Eagle’s medium (DMEM; 4.5 g/L glucose), supplemented with 10% fetal bovine serum (FBS), 100 U/mL penicillin and 100 μg/mL streptomycin at 37 °C in a humidified incubator with 95% air and 5% CO_2_ [30].

Cells were seeded in multi-well plates and serum deprived in DMEM supplemented with 1% fetal bovine serum, 100 U/mL penicillin and 100 μg/mL streptomycin for 16 h. Then, cells were stimulated with 100 µM of isoproterenol (Sigma Aldrich, Barcelona, Spain) or with 100 µM of deferoxamine (Sigma Aldrich, Spain) for 24 h. ISO and DEF concentrations were selected based on the literature [31,32,33,34] and further refined through our own dose–response and MTT viability assays, which defined the final experimental conditions (Supplementary Figure S1a–c) (*n* = 6–7 per experimental group, corresponding to six independent biological experiments).

### 2.2. Cell Viability (MTT Assay)

H9c2 cells were seeded in 96-well plates at a density of 4 × 10^3^ cells/well and treated with ISO, DEF, or ISO + DEF at the indicated concentrations and time points. Cell viability was assessed using the MTT reduction assay. Briefly, 10% (*v*/*v*) of MTT stock solution (5 mg/mL) was added to each well and incubated for 2 h at 37 °C. The medium was then removed and replaced with 100 µL of dimethyl sulfoxide (DMSO). Absorbance was measured at 490 nm in a microplate reader. Data are expressed as percentage relatives to untreated control cells (100%).

### 2.3. RNA Preparation and Quantitative Real-Time Reverse Transcription Polymerase Chain Reaction (RT-PCR) Analysis

mRNA levels were determined by RT-PCR. Total RNA was isolated from H9c2 cells using a Nucleospin RNA II kit (Macherey–Nagel, Zaragoza, Spain). RNA was quantified by a NanoDrop 1000 Spectrophotometer (Thermo Scientifc, Madrid, Spain). mRNA was transcribed into cDNA using a High-Capacity cDNA Reverse Transcription Kit (AppliedBiosystems, Foster City, CA, USA). TaqMan expression assays-on-demand (Thermo Scientific, Madrid, Spain) were used for rat divalent metal transporter 1 (*Dmt1*) (Rn01533109_ m1), rat transferrin receptor (*Tfrc*) (Rn01474701_m1), rat hepcidin antimicrobial peptide (Hamp) (Rn00584987_m1), rat ferroportin (*Fpn*) (Rn00591187_m1), rat iron regulatory protein 1 (*Aco1*) (Rn00569045_m1), rat iron regulatory protein 2 (*Ireb2)* (Rn00575852_m1), rat mitochondrial ferritin (*Ftmt*) (Rn01492073_s1), rat ferritin light chain 1 (*Ftl1*) (Rn04341729_g1), rat ferritin heavy chain 1 (*Fth1*) (Rn00820640_g1), rat mitoferrin 1 (*Mfrn1*) (Rn01753423_m1), rat mitoferrin 2 (*Mfrn2*) (Rn01411393_m1), rat ATP-binding cassette subfamily B member 7 (*Abcb7*) (Rn01497247_m1), rat ATP-binding cassette subfamily B member 8 (*Abcb8*) (Rn01442319_m1), rat mitofusin 1 (*Mfn1*) (Rn0594496_m1) and rat mitofusin 2 (*Mfn2*) (Rn01639199_m1).

Rat glyceraldehyde-3-phosphate dehydrogenase (*Gapdh*) was used as the endogenous control (Rn01775763_g1). The results were normalized to *Gapdh*, and relative quantification was performed using the comparative Ct (2-DDCt) method. mRNA levels were expressed as fold induction over control.

### 2.4. Immunoblotting

Whole protein extracts were collected from H9c2 cells in RIPA buffer (0.1% SDS, 150 mM NaCl, 1% Nonidet P40, 50 mM Tris–HCl, and 0.5% deoxycholate) containing phosphatase and protease inhibitors (Roche Diagnostics; Basel, Switzerland). Western blot analyses were performed using antibodies against TFRC, FERRITIN, HAMP, FTMT and ACTIN from Abcam (Cambridge, UK); FPN1, IRP1 (Iron regulatory protein 1), IRP2 (Iron regulatory protein 2), MFRN1, MFRN2 and DMT1 from Thermo Scientific (Spain); and MFN1 and MFN2 from SelleckChem (Tarragona, Spain). Detection was performed using the appropriate horseradish peroxidase (HRP)-conjugated secondary antibody (Dako; Glostrup, Denmark). The bands were visualized using Clarity^TM^ Western ECL Substrate (BioRad, Madrid, Spain) with the Quantity One 4.6.6. software (BioRad, Spain). The results were normalized to ACTIN, and the differences in the protein levels were expressed as percentage induction over control. Western blot original images can be found in Supplementary Materials (File S1).

### 2.5. Intracellular Iron Determination

The intracellular iron ions content was determined in H9c2 cells using the colorimetric Iron Assay kit (Abcam, Spain), following the manufacturer’s instructions. All assays were performed in duplicate and measured on a BioTek PowerWave XS microplate reader. Levels of total iron were expressed relative to untreated controls.

### 2.6. Analysis of Cell Bioenergetics (Seahorse XFe96)

ATP production, the oxygen consumption rate (OCR), the extracellular acidification rate (ECAR) and the proton efflux rate (PER) were measured using a Seahorse XFe96 Analyzer (Agilent Technologies, Santa Clara, CA, USA) with a Seahorse XF Real-Time ATP Rate Assay Kit (Agilent Technologies, USA) and a Seahorse XF Cell Mito Stress Test kit (Agilent, USA), following the manufacturer’s instructions. Seahorse XF Cell Mito Stress Assay Kit inhibitors were used in the following order, after measuring parameters at basal condition, Oligomycin, FCCP and Rotenone/Antimycin A. Parameters were calculated as follows: proton leak as the minimum rate measurement after Oligomycin injection extracting the non-mitochondrial oxygen consumption; maximal respiration as the maximum rate measurement after FCCP injection extracting non-mitochondrial oxygen consumption; non-mitochondrial oxygen consumption as the minimum rate measurement after Rotenone/Antimycin A injection; ATP-production coupled respiration as the last rate measurement before Oligomycin injection extracting the minimum rate measurement after Oligomycin injection; spare respiratory capacity (%) is derived from dividing maximal respiration by basal or induced respiration multiplied by 100. Briefly, H9c2 cells were seeded in 96-well Seahorse assay plates and were incubated at room temperature for 1 h. Then, the cells were cultured and treated as stated before. Before the assay, cells were washed once and then incubated for 1 h at 37 °C in a humidified incubator with 0% CO_2_, both with Seahorse XF DMEM (Agilent Technologies, USA) supplemented with 20 mM glucose, 2 mM L-glutamine and 1 mM sodium pyruvate. Hoechst staining was employed to quantify cell numbers for well-to-well normalization.

### 2.7. Statistical Analysis

Data are expressed as the mean ± standard error of the mean (SEM). Significant differences were established using Student’s *t*-test or one-way analysis of variance (ANOVA), followed by Bonferroni’s post hoc test, as appropriate. Data were analyzed by using the GraphPad Instat program 8.01 (GraphPad Software Inc., Boston, MA, USA). Differences were considered statistically significant at a *p*-value < 0.05.

## 3. Results

### 3.1. Isoproterenol Decreases Intracellular Iron Levels in Cardiac Cells

**Figure 1:**
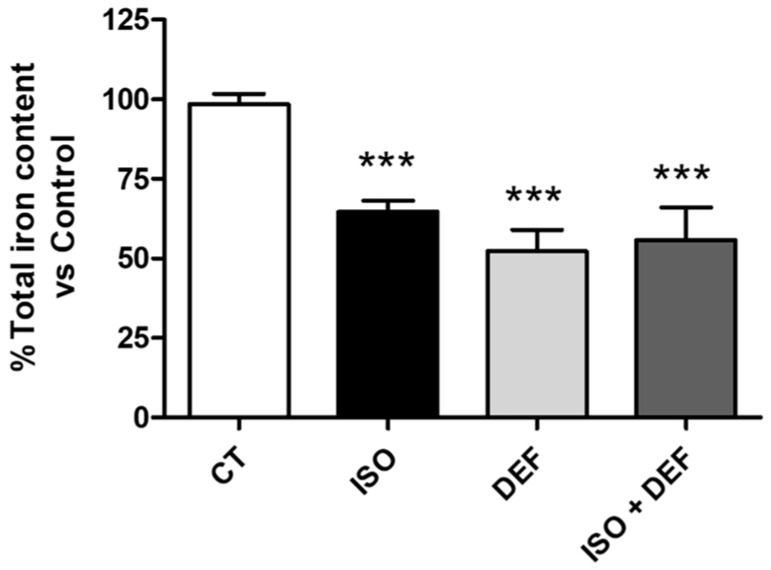
Isoproterenol depletes intracellular iron in H9c2 cells. Analysis of intracellular iron levels from the cells challenged with isoproterenol and/or deferoxamine. Data are expressed as mean ± SEM; (*** *p* < 0.001 vs. control).

First, intracellular iron levels were assessed in cells treated with ISO or/and DEF to compare the effects of the different stimuli. It was shown that ISO induced a reduction in intracellular iron levels similar to that caused by the specific iron chelator, DEF (Figure 1). Nevertheless, no synergistic effect was observed in cells exposed to both stimuli simultaneously.

### 3.2. Isoproterenol Induces Similar Changes to Deferoxamine in Most Intracellular Iron Metabolism Molecules in H9c2 Cells

**Figure 2:**
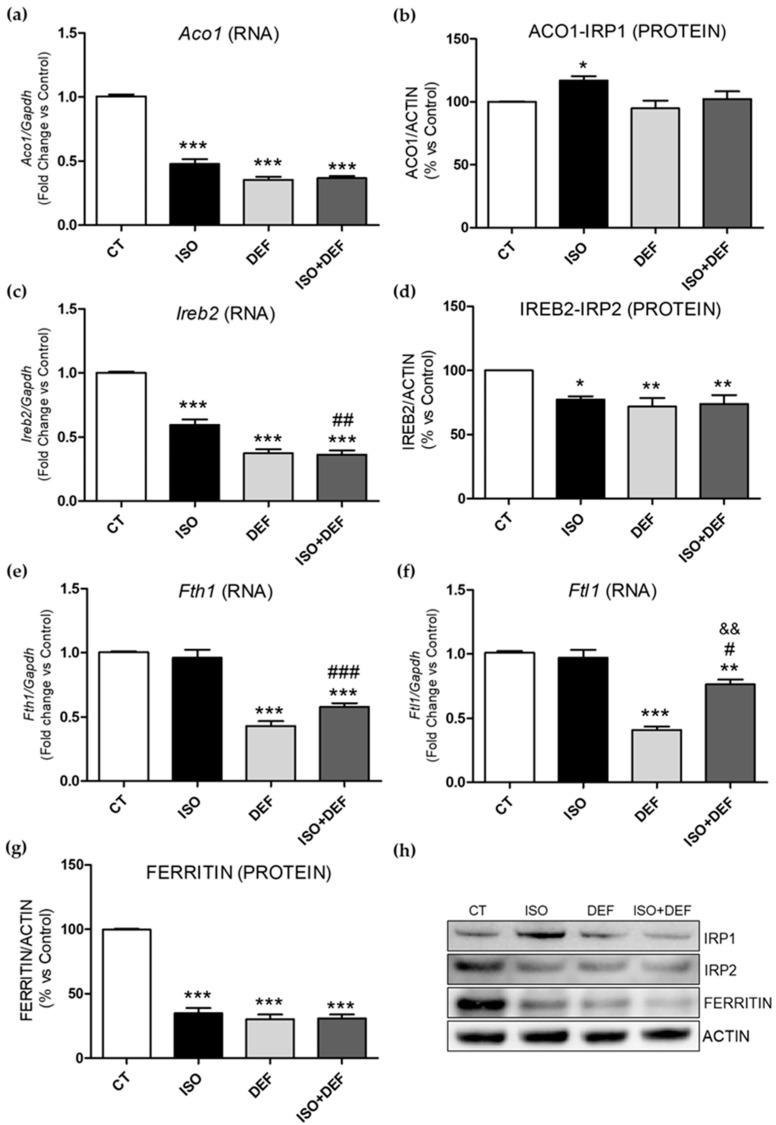
Isoproterenol modifies iron metabolism-related molecules in H9c2 cells. Analysis of mRNA (**a**,**c**,**e**,**f**) and protein levels (**b**,**d**,**g**,**h**) of IRP1 (*Aco1*) (**a**,**b**), IRP2 (*Ireb2*) (**c**,**d**), *Fth1* (**e**), *Ftl1* (**f**) and FERRITIN (**g**). Representative Western blot image of all the analyzed proteins (**h**). Western blot original images can be found in File S1. Data were normalized by the *Gapdh* mRNA or by ACTIN protein levels and expressed as mean ± SEM; (* *p* < 0.05, ** *p* < 0.01 and *** *p* < 0.001 vs. control; ^#^ *p* < 0.05, ^##^ *p* < 0.01 and ^###^ *p* < 0.001 vs. ISO; ^&&^ *p* < 0.01 vs. DEF).

Both the mRNA and protein levels of the main molecules involved in cellular iron uptake, release and storage were analyzed in the cells challenged with ISO, DEF and ISO + DEF. Firstly, two iron regulatory proteins (*Irp1* and *2*), involved in the translation of several iron metabolism proteins, were explored. *Irp1* (*Aco1*) (Figure 2a) and *Irp2* (*Ireb2*) (Figure 2c) mRNA levels were downregulated with all the stimuli. Accordingly, protein levels of IRP2 (Figure 2b,h) were reduced under all conditions, whereas IRP1 protein levels were increased under ISO conditions, with no changes observed in DEF/DEF + ISO-challenged cells (Figure 2d,h). Secondly, the cytoplasmic iron storage molecule ferritin, encoded by ferritin heavy chain 1 (*Fth1*) and ferritin light chain 1 (*Ftl1*), was analyzed. While *Ftl1* (Figure 2e) and *Fth1* (Figure 2f) mRNA levels remained unchanged in the ISO-stimulated cells, the expression of both ferritin chains was reduced in the cells challenged with DEF. Conversely, FERRITIN protein levels decreased under all experimental conditions (Figure 2g,h). The combination of both stimuli did not show a synergistic effect.

**Figure 3:**
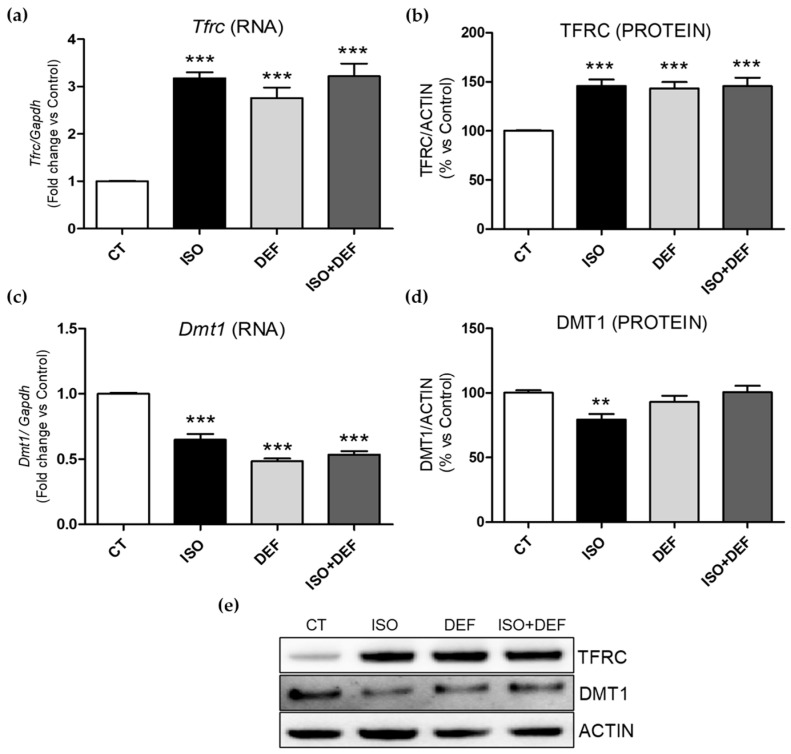
Isoproterenol modifies molecules involved in intracellular iron uptake in H9c2 cells. Analysis of mRNA (**a**,**c**) and protein (**b**,**d**,**e**) levels of TFRC (**a**,**b**) and DMT1 (**c**,**d**). Representative Western blot image of all the analyzed proteins (**e**). Western blot original images can be found in File S1. Data were normalized by the Gapdh mRNA or ACTIN protein levels and expressed as mean ± SEM; (** *p* < 0.01 and *** *p* < 0.001 vs. control).

**Figure 4:**
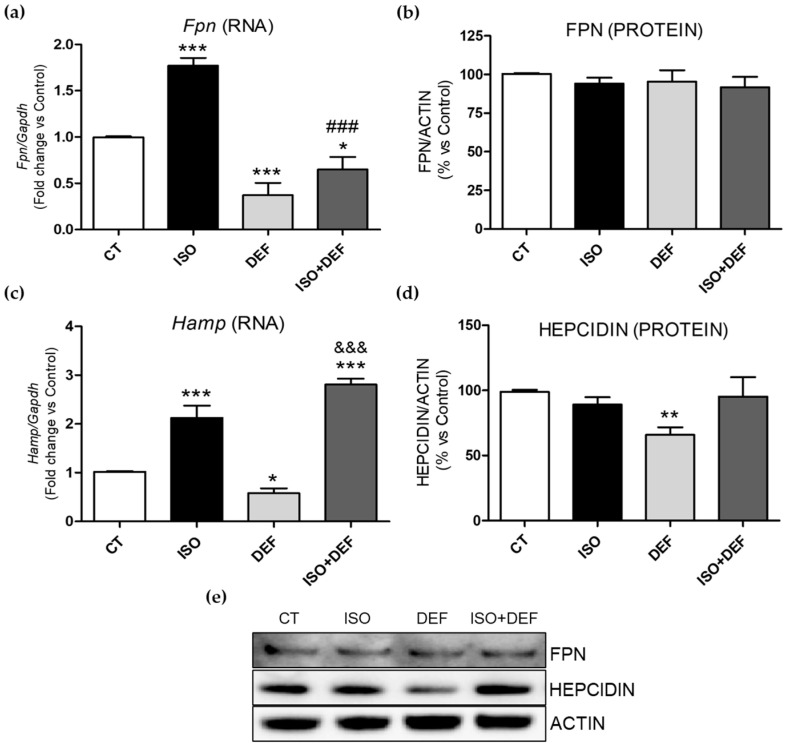
Isoproterenol modifies molecules involved in intracellular iron release in H9c2 cells. Analysis of mRNA (**a**,**c**) and protein levels (**b**,**d**,**e**) of FPN (**a**,**b**) and HEPCIDIN (**c**,**d**). Representative Western blot image of all the analyzed proteins (**e**). Western blot original images can be found in File S1. Data were normalized by the Gapdh mRNA or ACTIN protein levels and expressed as mean ± SEM; (* *p* < 0.05, ** *p* < 0.01 and *** *p* < 0.001 vs. control; ^###^ *p* < 0.001 vs. ISO and ^&&&^ *p* < 0.001 vs. DEF).

Next, the levels of the main iron transporters into the cell, transferrin receptor (Tfrc) and divalent metal transporter 1 (Dmt1), were also determined. While *Tfrc* expression was increased under all conditions studied (Figure 3a), *Dmt1* mRNA levels were decreased (Figure 3c). Accordingly, similar changes were found in TFRC protein levels (Figure 3b,e), but a significant decrease in DMT1 protein levels was observed only in cells challenged with ISO (Figure 3d,e). In order to look deeper into the role of the molecules related to the cellular iron release, ferroportin (Fpn) and its inhibitor, hepcidin antimicrobial peptide (*Hamp*), were analyzed. While mRNA levels of both molecules were upregulated in the ISO-stimulated cells, DEF reduced the expression of both genes (Figure 4a,c). In the combined condition, *Fpn* levels resembled those observed with DEF, whereas *Hamp* expression was similar to the increase seen with ISO alone (Figure 4a,c). Regarding protein levels, FPN remained unchanged (Figure 4b,e), and a significant decrease in HEPCIDIN was detected only in the DEF-stimulated cells (Figure 4d,e).

### 3.3. Regulation of Mitochondrial Iron Metabolism Molecules in ISO- and/or DEF-Challenged Cardiac Cells

**Figure 5:**
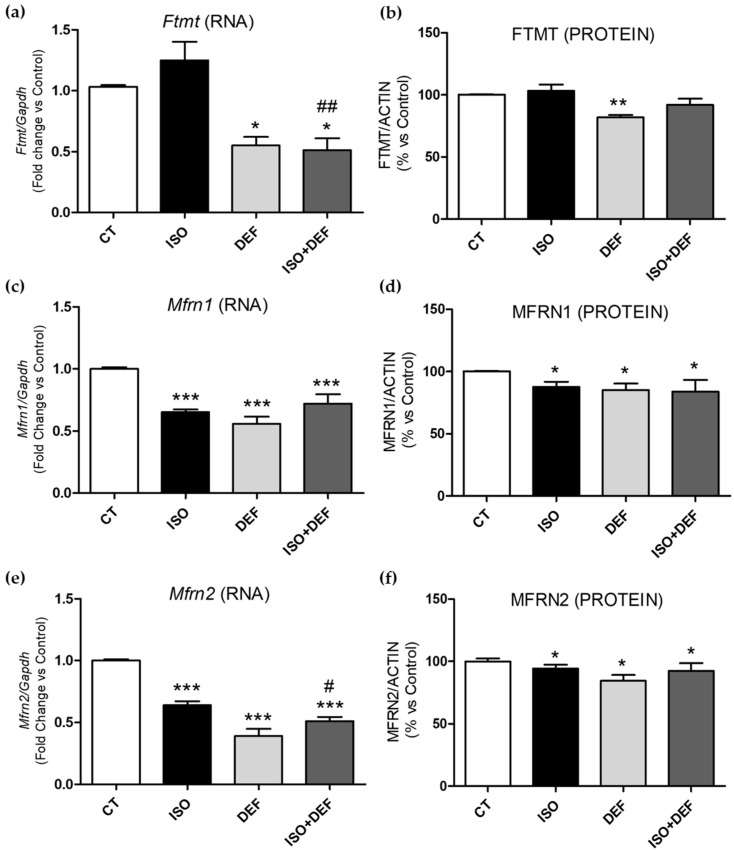

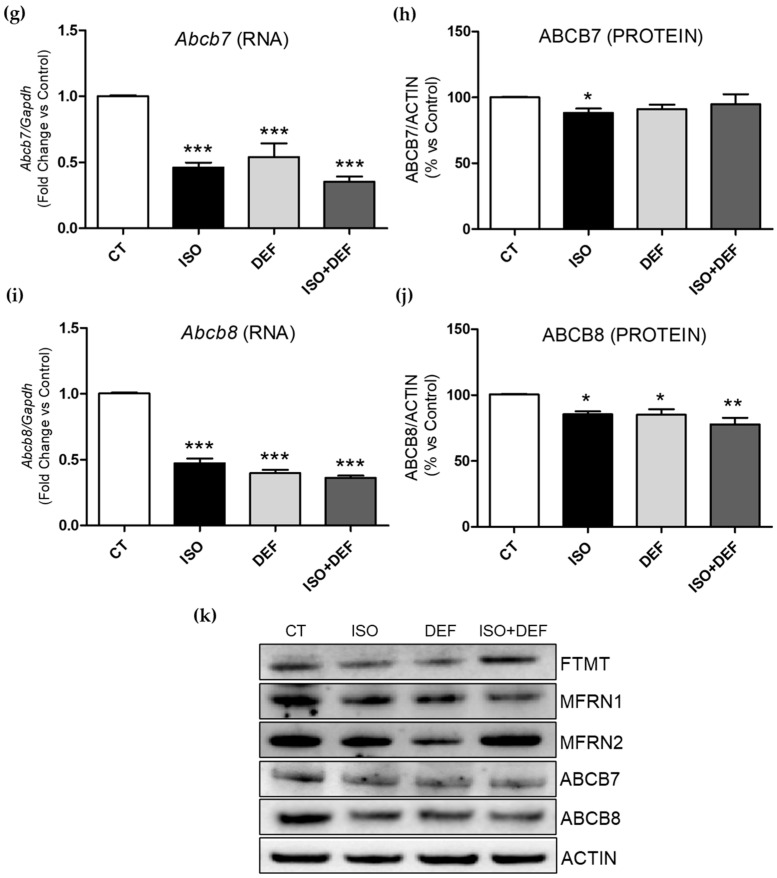
Isoproterenol modifies the expression of molecules involved in mitochondrial iron uptake and release in H9c2 cells. Analysis of *Ftmt, Mfrn1, Mfrn2, Abcb7* and Abcb8 mRNA (**a**,**c**,**e**,**g**,**i**) and protein levels (**b**,**d**,**f**,**h**,**j**). Representative Western blot image of all the analyzed proteins (**k**). Western blot original images can be found in File S1. Data were normalized by the Gapdh mRNA or by ACTIN protein levels and expressed as mean ± SEM; (* *p* < 0.05, ** *p* < 0.01 and *** *p* < 0.001 vs. control; ^#^ *p* < 0.05 and ^##^ *p* < 0.01 vs. ISO).

Due to the important role of iron in mitochondrial function, the mitochondrial iron storage molecule mitochondrial ferritin (Ftmt), the mitochondrial iron uptake transporters Mitoferrin 1 (Mfrn1) and 2 (Mfrn2), and the mitochondrial iron release molecules ATP-binding cassette subfamily B member 7 (Abcb7) and 8 (Abcb8) were analyzed. *Ftmt* mRNA levels were not significantly modified when the cells were challenged with ISO, but its expression was downregulated with DEF and ISO + DEF (Figure 5a). The expression of both mitochondrial iron import and export molecules decreased in all the conditions studied (Figure 5c,e,g,i). The protein levels of the molecules showed a different pattern depending on the stimuli. FTMT levels were unchanged with ISO and ISO + DEF and decreased significatively with DEF (Figure 5b,k). MFRN1, MFRN2 and ABCB8 levels significatively decreased in all the analyzed conditions (Figure 5d,f,j,k). ABCB7 (Figure 5h,k) levels decreased in general, but only significatively with ISO.

### 3.4. Isoproterenol Triggers Hypertrophy and Impairs Mitochondrial Function in H9c2 Cells

**Figure 6:**
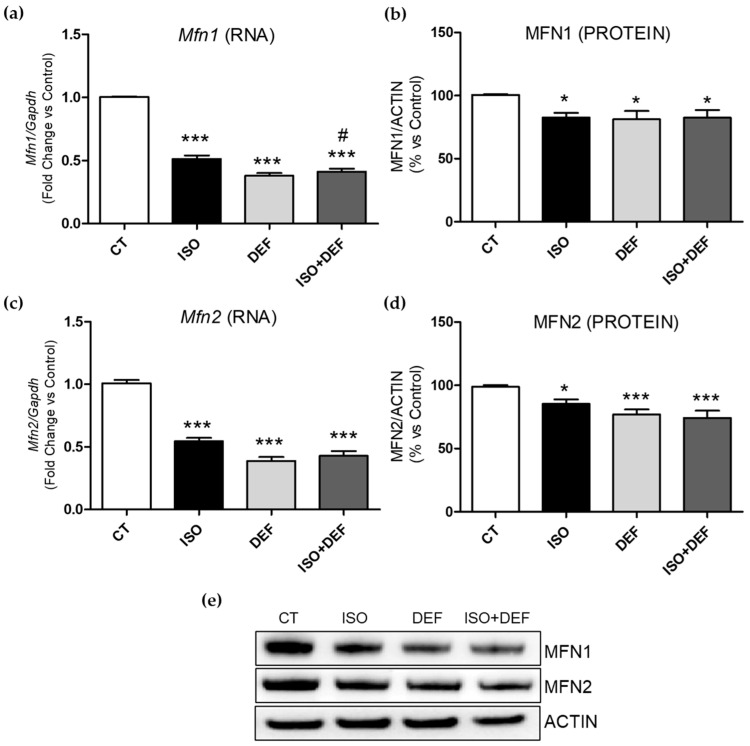
Isoproterenol downregulates mitofusin 1 and 2 expressions in H9c2 cells. Analysis of *Mfn1* and *Mfn2* mRNA (**a**,**c**) and protein levels (**b**,**d**). Representative Western blot image of all the analyzed proteins (**e**). Western blot original images can be found in File S1. Data were normalized by the *Gapdh* mRNA or ACTIN protein levels and expressed as mean ± SEM; (* *p* < 0.05 and *** *p* < 0.001 vs. control; ^#^ *p* < 0.05 vs. ISO).

Several parameters were assessed to characterize mitochondrial function in cardiac cells. Mitofusin 1 (Mfn1) and mitofusin 2 (Mfn2) are mitochondrial outer membrane GTPases that regulate mitochondrial fusion, essential for maintaining mitochondrial function and cellular energy balance. Furthermore, their expression is often reduced in models of cardiac hypertrophy. In the present study, both the mRNA and protein levels of MFN1 (Figure 6a,b,e) and MFN2 (Figure 6c,d,e) were downregulated across all experimental conditions.

**Figure 7:**
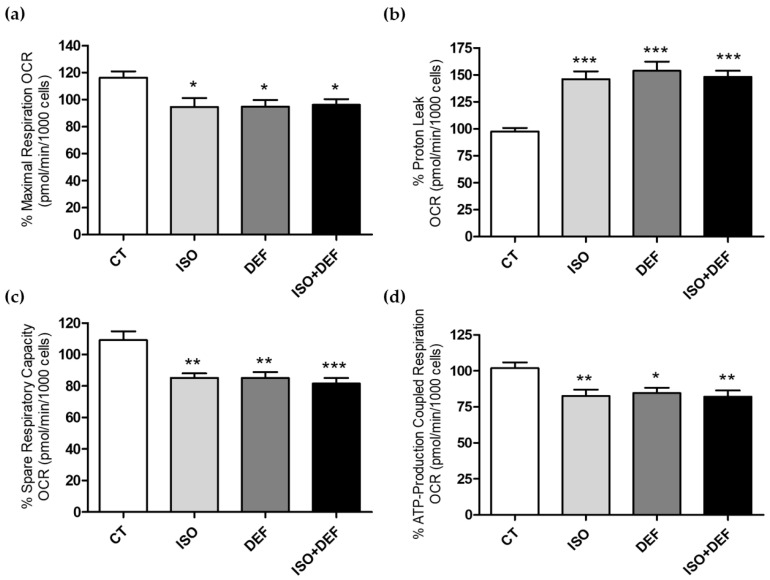
Isoproterenol impairs mitochondrial function. Quantification of different mitochondrial parameters using Seahorse Analyzer tests. The % maximal respiration (OCR) (**a**), the % proton leak (OCR) (**b**), the % spare respiratory capacity (OCR) (**c**) and the % ATP-production coupled respiration (OCR) (**d**) are shown. The data are expressed as mean ± SEM; (* *p* < 0.05, ** *p* < 0.0 and *** *p* < 0.001 vs. control).

The analysis of mitochondrial impairment and energy state was analyzed by Seahorse technology. Results related to mitochondrial impairment, reduced maximal respiration, spare respiratory capacity and ATP-production coupled respiration (Figure 7a,c,d), together with increased proton leak (Figure 7b), were obtained in all the experimental conditions.

**Figure 8:**
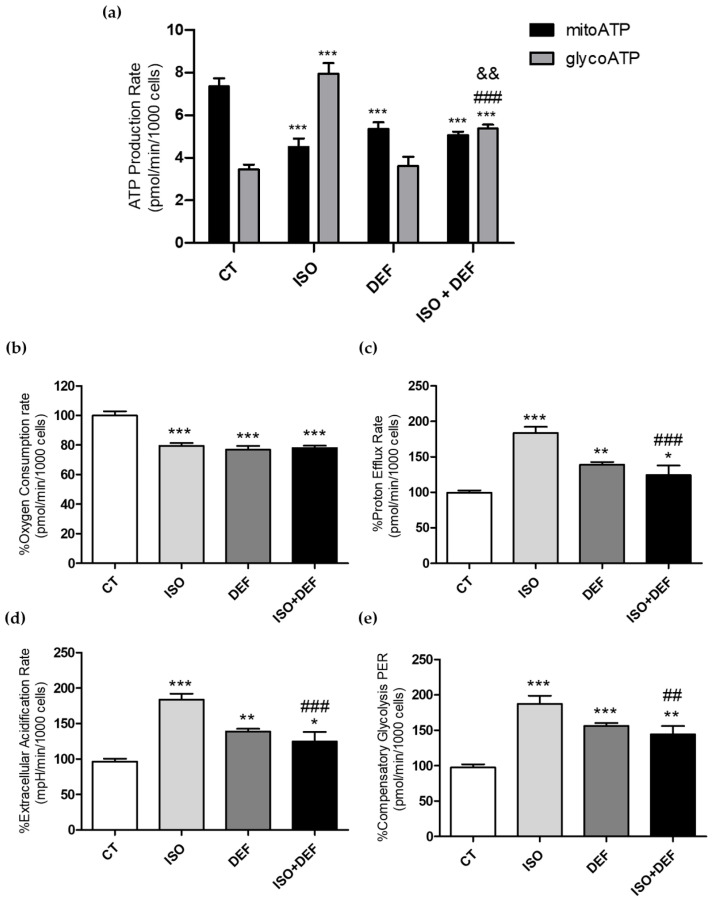
Isoproterenol impairs mitochondrial function and bioenergetic state. Quantification of different mitochondrial parameters using Seahorse Analyzer tests. The ATP production rate (mitoATP and glycoATP) (**a**), the % oxygen consumption rate (OCR) (**b**), the % proton efflux rate (PER) (**c**), the % extracellular acidification rate (ECAR) (**d**) and % compensatory glycolysis PER (**e**) are shown. ATP production was expressed by pmol/min/1000 cells and the rest of the data are expressed as mean ± SEM; (* *p*< 0.05, ** *p* < 0.0 and *** *p* < 0.001 vs. control; ^##^ *p* < 0.01 and ^###^ *p* < 0.001 vs. ISO; ^&&^ *p* < 0.01 vs. DEF).

Oxidative phosphorylation (OXPHOS) is critical for cardiomyocytes because it provides most of the ATP required for cardiac contraction (mitoATP). When the cells were challenged with ISO, there was a dramatic change in their energy metabolism, increasing their energy demand and switching to glycolysis as the dominant ATP-generating pathway (glycoATP) (Figure 8a). However, with DEF, the cellular energy metabolism followed a pattern like that of the controls, although mitoATP generation notably diminished (Figure 8a). Under the combined condition, we observed an intermediate response, with mitoATP remaining reduced and glycoATP showing a modest increase compared with the control cells (Figure 8a).

When the other mitochondrial functional parameters were assessed, a reduction in oxygen consumption rate (OCR) was observed across all conditions (Figure 8b). Conversely, the proton efflux rate (PER) (Figure 8c) and extracellular acidification rate (ECAR) (Figure 8d) increased in the experimental groups, with the greatest elevation occurring in the ISO-challenged cells. Finally, compensatory glycolysis PER was upregulated under both stimuli, showing a stronger response in ISO-challenged cells (Figure 8e).

## 4. Discussion

ID is strongly linked to adverse outcomes in HF [35,36], yet it remains unclear whether cardiomyocyte ID is merely a consequence of HF or an active driver of cellular dysfunction that led to disease progression [22]. Our results show that β-adrenergic stimulation (as one of the major components of neurohormonal activation) disrupts iron handling and triggers intracellular ID, partially mimicking the effects of iron chelation in cardiac cells. This alteration is accompanied by a metabolic shift toward glycolytic ATP production and mitochondrial impairment. While the clinical benefits of intravenous iron have often been attributed to peripheral effects [18,37], emerging evidence [38,39] and the present findings support a direct cardiac contribution. Together, our findings highlight intracellular ID is an important component of the cellular response to β-adrenergic stimulation in cardiac cells.

The present study used the in vitro cardiac cell line H9c2 [40,41]. This cell line offers a robust and ethically aligned in vitro model for cardiac mitochondrial research, with metabolic and mitochondrial characteristics that more closely resemble primary cardiomyocytes than those of other cardiac cell lines [40]. All experiments were conducted using this model to avoid the variability introduced by metabolically divergent cell lines. In our work, H9c2 cells were challenged with the β1/β2-adrenergic receptor agonist ISO, and the iron chelator DEF. ISO, which induces myocardial stress and mitochondrial damage, is extensively used for in vitro and in vivo studies to test the efficacy of therapeutic strategies in HF [6,42]. The comparative analysis of DEF and ISO enables discrimination between the effects on iron metabolism caused by β-adrenergic activation and those resulting from specific iron chelation, in which the response is driven exclusively by metal depletion.

This approach provides insight into potential metabolic pathways related to β-adrenergic stimulation and underscores the important role of iron in cardiac cell pathophysiology.

Previous studies have reported iron depletion in the myocardial tissue of patients with HF [23,28]. Additionally, our group showed that neurohormonal activation is capable of triggering intracellular iron depletion in cardiac cells [30]. Our present findings reveal that both the iron chelator and the β-adrenergic agonist induce a reduction in intracellular iron levels. To assess whether the iron chelator could potentiate the effect of ISO, iron levels were also measured when both stimuli were applied simultaneously. However, no synergistic effect was observed, possibly because stimulation with either agent alone had already achieved the maximum effect with respect to iron levels. Nevertheless, to better evaluate whether β-adrenergic stimulation and iron chelation act through convergent, additive, or independent mechanisms, we examined all molecular markers and functional outcomes under the combined condition.

In cardiac cells, ID may arise through multiple mechanisms involving disruptions in iron uptake, release, and storage pathways. The present study examined several of the key molecular pathways previously linked to cellular ID in the context of HF [27]. Irps modulate the translation of key molecules involved in these processes, adjusting their activity according to intracellular iron availability [43], and their deletion in cardiomyocytes leads to cellular ID [44]. Haddad et al. showed that Irps are essential for iron delivery to cardiomyocytes and HF prevention in mice, and that Irp activity is reduced in patients with HF, in association with myocardial iron depletion [26]. In line with these findings, our data showed a downregulation of *Irp* in cardiac cells under all stimuli, suggesting that Irps in ISO-treated cells may respond in a manner compatible with iron-depleted conditions. This suggests that one of the key regulators of iron metabolism is transcriptionally altered in response to β-adrenergic activation in cardiac cells. On the other hand, our study did not show changes in the expression of the cytoplasmic iron storage molecule, ferritin, in response to ISO stimuli, but instead a decrease in its protein levels. This may be related to an increased cellular demand for iron, promoting ferritin iron release without initially affecting transcription. Previous studies showed a similar pattern in myocardial infarction and a HF mouse model, in which ferritin protein decreased while gene expression remained stable [45]. In contrast, both ferritin mRNA and protein levels were reduced in the DEF and ISO + DEF conditions, consistent with low-iron environments in which cells attempt to restore intracellular iron levels [45]. In this context, it is important to consider processes such as ferritin turnover, ferritinophagy, and the dynamic regulation of intracellular iron pools, which could also explain the decrease in ferritin protein levels [46,47]. Accumulating evidence indicates that ferritinophagy plays a significant role in cardiac responses to β-adrenergic stimulation. Recently, Deng et al. demonstrated that ISO activates NCOA4-dependent ferritinophagy in cardiomyocytes, contributing to iron dysregulation and subsequent cellular injury [48,49]. Furthermore, different studies emphasize that alterations in intracellular iron handling, including ferritin dynamics, mitophagy, and the regulation of intracellular iron pools, are important components of cardiac iron homeostasis [50,51].

Next, we assessed the levels of TFRC involved in the uptake of transferrin-bound iron [45]. Our results showed *Tfrc* upregulation under all stimuli, indicating that H9c2 cells were in an iron-depleted environment induced by ISO or DEF, and suggesting transcriptional reprogramming aimed at enhancing iron uptake. Finally, although Dmt1 was downregulated under both stimuli, we did not observe protein alterations in cells challenged with DEF or ISO + DEF. Since Dmt1 mediates non-transferrin-bound iron uptake [52], Dmt1 upregulation could act as a compensatory mechanism of the Tfrc-mediated iron import [53]. Therefore, the marked increase in *Tfrc* expression in ISO-treated cells may have driven a downregulation of *Dmt1* to prevent iron toxicity. Furthermore, it is still debated whether the expression of *Dmt1* changes with the concentration of iron in the heart [27,54].

Neurohormonal stimuli also modulate the molecules involved in cellular iron efflux, Fpn and hepcidin. In the heart, Hamp contributes to local iron homeostasis by inhibiting Fpn, the exporter responsible for releasing intracellular iron [55]. Dysregulation of the Fpn/Hamp axis can lead to either iron overload or anemia, depending on the direction of the functional imbalance [56]. Under the stimuli studied, we found different patterns in the expression of the iron export molecules. While ISO stimulation led to an upregulation of both *Fpn* and *Hamp* mRNA, DEF resulted in a reduction in the expression of these genes in cardiac cells. As is often observed in the combined condition, the overall response was intermediate; however, in this case, DEF predominantly shaped the transcriptional pattern of *Fpn*, while ISO primarily influenced *Hamp* expression. Regarding protein levels, only HEPCIDIN showed a significant reduction under DEF exposure. In this context, the downregulation of *Fpn* would act as a protective mechanism, making additional regulation via hepcidin unnecessary. Conversely, the increase in *Fpn* expression in ISO-challenged cells may indicate an alteration in iron metabolism triggered by β-adrenergic receptor activation. This could explain, on the one hand, part of the decrease in intracellular iron levels and, on the other hand, the increase in *Hamp*, as a mechanism to prevent, or to be prepared to prevent, the release of iron from the cell. The mechanisms that involve the delivery of iron from the intracellular environment to FPN are not well understood. Furthermore, an increase in *Hamp* expression is commonly observed in organs under stress, including the heart. During the early phases of myocardial infarction and myocarditis, cardiac *Hamp* expression increased significantly, and this upregulation is associated with inflammatory markers [57]. Our data suggest that, while ISO-stimulated cells attempted to activate pathways promoting intracellular iron retention by increasing *Hamp* levels, exposure to the β-adrenergic agonist instead led to an abnormal increase in *Fpn*, which may favor iron release and thereby contribute to ID.

Altogether, our findings indicate that β-adrenergic stimulation may promote intracellular iron depletion by disrupting both intracellular iron release and extracellular iron uptake in cardiac cells, although further research is warranted to unveil the upstream signaling pathway linking β-adrenergic stimulation to the initial dysregulation of *Fpn, Hamp* and *Dmt1* expression.

The contribution of iron to cardiac function is tightly linked to mitochondria, given its essential role as an enzymatic cofactor. Moreover, mitochondrial dysfunction is increasingly recognized as a key driver of HF [58,59]. Intracellularly, iron is required by mitochondria (the center of iron utilization) to maintain their normal physiological function [60]. The heart continuously consumes large amounts of energy, predominantly for contraction and ion transport, yet its capacity for energy storage is unexpectedly low [61]. Mitochondria produce more than 95% of the ATP in the myocardium, and play critical roles in regulating redox status, calcium handling and lipid biosynthesis. It is therefore not surprising that mitochondrial dysfunction has been strongly associated with the development of cardiomyopathy and an increased risk of HF [61,62].

Therefore, given the importance of iron in maintaining proper mitochondrial function, we evaluated the status of several molecules involved in mitochondrial iron uptake, release, and storage. Regarding storage, no differences were observed in *Ftmt* expression or protein levels when comparing the effect of ISO to the control cells; however, DEF and ISO + DEF induced a downregulation of this molecule. Thus, β-adrenergic activation does not appear to affect the mitochondrial iron storage molecule. Regarding mitochondrial iron uptake, the expression and the protein levels of MFRN1 and MFRN2 were downregulated under all experimental conditions. Paradkar et al. showed that the reduction in *Mfrn1* and *Mfrn2* expression through RNA interference resulted in decreased mitochondrial iron accumulation, heme synthesis and iron–sulfur cluster synthesis [63]. In addition, previous studies have shown that the increased expression of *Mfrn1* requires *Irp2*, although no direct interactions between them have been identified [64,65]. Therefore, the downregulation of *Irp2* observed across all experimental conditions could be associated with the concomitant reduction in *Mfrns* expression. Nevertheless, several studies indicate that Mfrn regulation is a complex process essential for mitochondrial iron homeostasis. It remains unclear whether Mfrns are controlled at the transcriptional, translational, or post-translational level, and how cellular or mitochondrial iron status influences their expression under different physiological or pathological conditions [65].

Finally, the analysis of the mitochondrial iron exporters *Abcb7* and *Abcb8* revealed a downregulation under all experimental conditions, also at the protein level; however, in this case, the decrease reached statistical significance only for ABCB8 and ABCB7 under DEF treatment. This pattern suggests that the cell attempts to minimize iron loss while simultaneously preventing excessive iron influx, thereby maintaining mitochondrial iron homeostasis as stable as possible. Previous studies showed that chronic high-pressure overload results in ABCB7 deficiency, contributing to mitochondrial dysfunction, metabolic alterations, and worsening cardiac function [66]. On the other hand, the ABCB8 transporter is important for maintaining normal heart function [67]. Its loss impairs left ventricular function and leads to cardiomyopathy [68]. In addition, as a half-transporter, ABCB8 may interact with ABCB7 to form a functional multimeric complex involved in iron transport [69].

A key hallmark of the failing heart is the dysregulation of mitochondrial dynamics. Typically, there is an increase in levels of fission-associated proteins (e.g., DRP1 [dynamin-related protein 1]) and a decrease in levels of fusion-associated proteins (e.g., MFN2 (mitofusin 2)) [70,71]. Furthermore, cardiac hypertrophy (an early and common feature of HF) is closely associated with mitochondrial dysfunction [72]. Several studies showed Mfn2 as a putative inhibitor of cardiac hypertrophy, reporting its downregulation across various experimental models of cardiac hypertrophy [73]. In addition, its upregulation inhibits Ang II-induced myocardial hypertrophy, indicating that Mfn2 is a critical regulator of myocyte hypertrophic remodeling [74]. Our present study showed a general decrease in the expression and protein levels of MFN1 and MFN2 under all conditions studied. These results are consistent with the fact that hypertrophy is caused both by β-adrenergic activation and by direct ID in cardiac cells [75]. On the other hand, the reduction in *Mfn* expression aligns with the impairment in mitochondrial function. The absence of either MFN1 or MFN2 leads to distinct types of mitochondria fragmentation. Cells lacking both MFN1 and MFN2 have severe cellular defects, including impaired growth, mitochondrial membrane potential heterogeneity, and reduced cellular respiration. Decreased expression of *Mfn2* promotes excessive mitochondrial fission, which has been linked to the development of HF in both rats and humans with pulmonary arterial hypertension [70]. Additionally, *Mfn1* is significantly reduced in non-responding HF patients, suggesting that its suppression, and the consequent disruption of mitochondrial dynamics, could be involved in the pathology of these patients [76].

Finally, overall mitochondrial function, and specifically ATP production, was assessed using Seahorse technology. Based on the parameters assessed in the Mito Stress Test, the treatment induced a general pattern consistent with moderate mitochondrial dysfunction. Specifically, the reduction in maximal respiration indicates the diminished ability of the respiratory chain to reach its full oxidative capacity, while the decrease in spare respiratory capacity suggests a reduced bioenergetic reserve and a lower ability of the cells to respond to increased energetic demands. In addition, the rise in proton leak and the decline in ATP-linked respiration point to decreased mitochondrial efficiency and impaired ATP production. Altogether, these changes support the presence of mitochondrial impairment under experimental conditions. In addition, the results showed a complete shift in ATP production in the cells challenged with ISO, characterized by a decrease in mitoATP (ATP derived from oxidative phosphorylation) and an increase in glycoATP (ATP produced via glycolysis). The reduction in mitoATP production induced by ISO and the shift toward glycolytic ATP reliance may be related to the strict bioenergetic specialization of cardiac tissue. Comparative transcriptomic studies show that the heart is highly enriched in nuclear-encoded mitochondrial genes involved in ATP synthase and OXPHOS [77,78]. Moreover, the tissue-specific expression of nuclear-encoded mitochondrial protein genes, particularly those linked to ATP synthase, highlights the tightly regulated mitochondrial architecture required to sustain cardiac energy homeostasis [79]. On the other hand, DEF treatment did not induce such a shift; instead, it resulted solely in a reduction in mitoATP levels. This may indicate that DEF does not elicit the metabolic stress that would drive an increase in ATP synthesis via glycolysis (and thus no rise in glycoATP), and a decrease in mitoATP due to the profound ID. With the combined condition, we observed that the individual effects of each compound were attenuated, resulting in an intermediate phenotype that reflects the partial contribution of both interventions. The present results are consistent with current knowledge of altered energy metabolism in HF. A characteristic feature of failing myocardium is a shift towards increased glucose utilization as the primary substrate for ATP generation [80,81]. The role of this metabolic shift is to minimize the oxidative damage occurring during Fatty Acid (FA) utilization, and to increase the ATP generation/oxygen consumption ratio [82]. Under physiological conditions, more than 95% of ATP in the heart is generated from oxidative phosphorylation, mainly via FA oxidation, with the remaining 5% produced by glycolysis. It is generally accepted that the utilization of glucose increases as HF progresses, accompanied by a concomitant reduction in FA oxidation [76]. Importantly, during HF, several energetic maladaptation mechanisms arise within myocardial tissue due to heightened sympathetic drive and an overactive renin–angiotensin–aldosterone system, which further amplify oxidative stress and contribute to impaired calcium handling [83]. Moreover, the excessive adrenergic signaling that accompanies the progression of HF [84] has a detrimental impact on the primarily adaptive shift toward glucose metabolism [82]. Myocardial ID in patients with HF may further promote glucose rather than FA utilization and, coupled with impaired protection against ROS, contribute to myocardial dysfunction and adverse remodeling. The notion that severe myocardial ID can cause mitochondrial dysfunction is supported by evidence showing that isolated cardiac ID induces mitochondrial respiratory dysfunction and fatal cardiomyopathy in mice [85]. Altogether, our results are in line with the data reviewed by Brown et al. suggesting that mitochondrial impairment may be a key driver of cardiomyocyte injury and HF progression [61].

Despite our data supporting the hypothesis that β-adrenergic stimulation directly impacts intracellular iron metabolism and mitochondrial function in cardiac cells, further research is warranted to validate our findings in more mature cardiomyocytes and cardiac systems, in order to better reproduce the physiology of adult cardiac tissue and to more precisely define iron metabolism in the context of HF.

## 5. Conclusions

Our data provide evidence that β-adrenergic stimulation lowers intracellular iron and impairs mitochondrial function in cardiac cells. The similar effects observed with the iron chelator suggest a key role for iron in the agonist-driven response. Conversely, the differences between both stimuli may reflect β-adrenergic-specific pathways capable of inducing ID independently of any pre-existing deficit. Finally, it is important to highlight that β-adrenergic activation triggers a metabolic shift characterized by reduced mitoATP and glycoATP production.

Taken together, the present findings suggest that ID may constitute a key contributor to the pathophysiological mechanisms mediated by β-adrenergic stimulation in cardiac cells.

## Data Availability

All relevant data are included in this published article.

## Supplementary Materials

The following supporting information can be downloaded at: https://www.mdpi.com/article/10.3390/biom16040582/s1, Figure S1: Dose–response MTT viability assay. File S1. original-images.

## Abbreviations

The following abbreviations are used in this manuscript:

ID	Iron Deficiency
HF	Heart Failure
SNS	Sympathetic Nervous System
ISO	Isoproterenol
DEF	Deferoxamine
HFrEF	Reduced Left Ventricle Ejection Fraction
ATP	Adenosine Triphosphate
ROS	Reactive Oxygen Species
Irp1	Iron Regulatory Protein 1
Irp2	Iron Regulatory Protein 2
Fth1	Ferritin Heavy Chain 1
Ftl1	Ferritin Light Chain 1
Tfrc	Transferrin Receptor
Dmt1	Divalent Metal Transporter 1
Fpn	Ferroportin
Hamp	Hepcidin Antimicrobial Peptide
Ftmt	Mitochondrial Ferritin
Mfrn1	Mitoferrin 1
Mfrn2	Mitoferrin 2
Abcb7	ATP-Binding Cassette subfamily B member 7
Abcb8	ATP-Binding Cassette subfamily B member 8
Mfn1	Mitofusin 1
Mfn2	Mitofusin 2
OXPHOS	Oxidative Phosphorylation
OCR	Oxygen Consumption Rate
PER	Proton Efflux Rate
ECAR	Extracellular Acidification Rate
DRP1	Dynamin-Related Protein 1
FAs	Fatty Acids
