# Supplementary material for: Iron Matters: Comparative Impact of Beta-Adrenergic Stimulation and Iron Chelation on Cardiac Iron Metabolism and Mitochondrial Function
Source: Biomolecules. 2026 Apr 14;16(4):582. doi: 10.3390/biom16040582 (PMC13113498; doi:10.3390/biom16040582)
Supplement: Supplementary file 1 [file biomolecules-16-00582-s001.zip › biomolecules-4195121-Figure S1.pdf]

Supplementary Figure S1

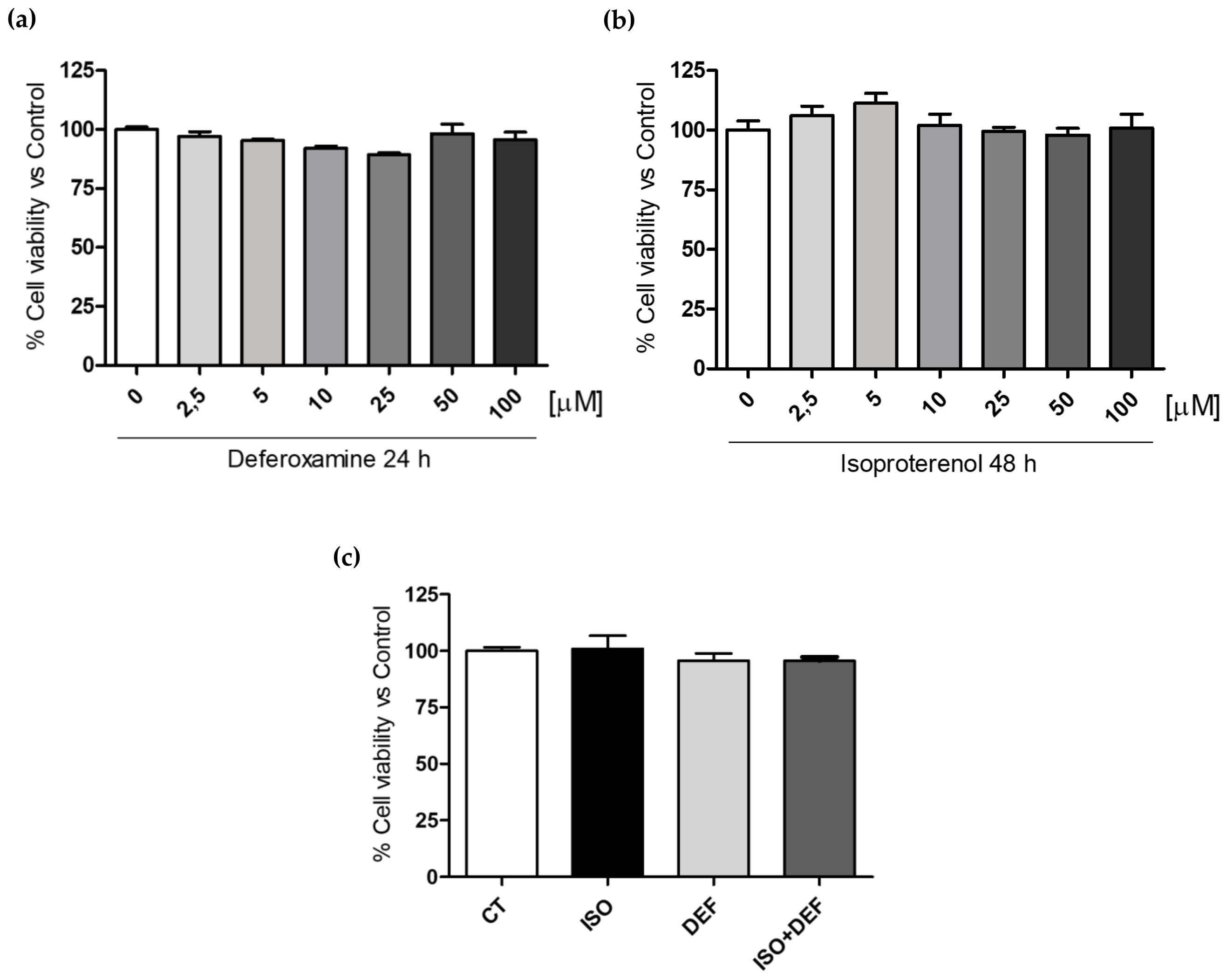

Supplementary Figure S1. Dose–response MTT viability assay. MTT analysis showing the response to different concentrations of Deferoxamine at 24 h (a) and Isoproterenol at 48 h (b) and different experimental conditions analyzed (c). Data are expressed as mean ± SEM
