# Supplementary figures and images for: Iron Matters: Comparative Impact of Beta-Adrenergic Stimulation and Iron Chelation on Cardiac Iron Metabolism and Mitochondrial Function
Source: Biomolecules. 2026 Apr 14;16(4):582. doi: 10.3390/biom16040582 (PMC13113498; doi:10.3390/biom16040582)

IRP1

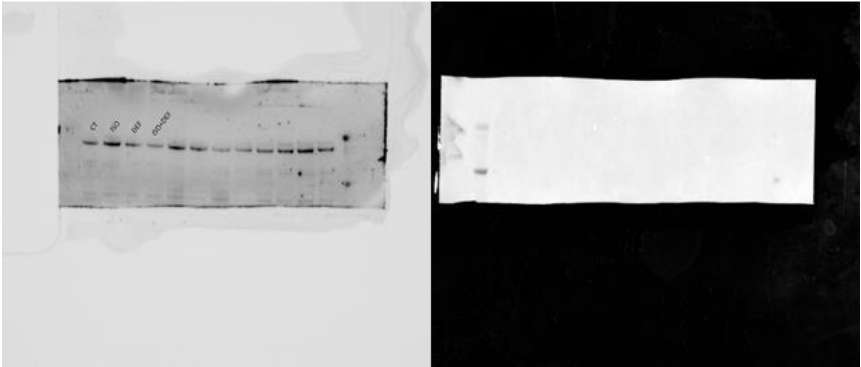

IRP2

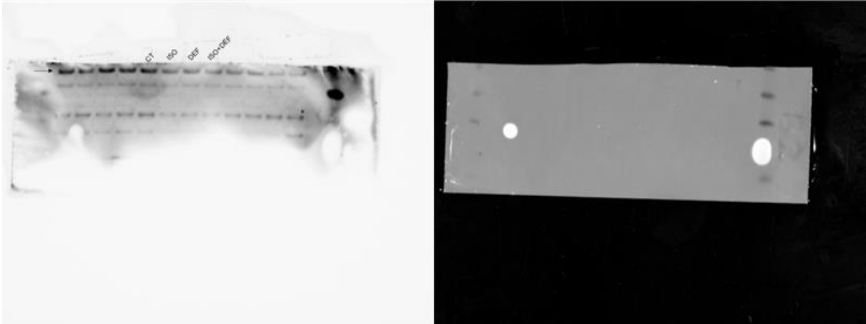

FERRITIN

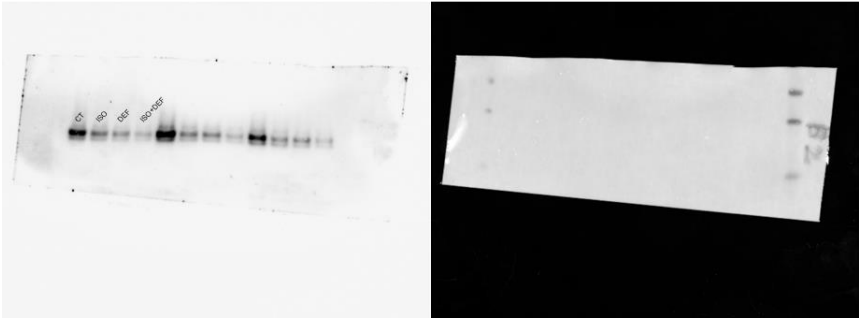

TFRC

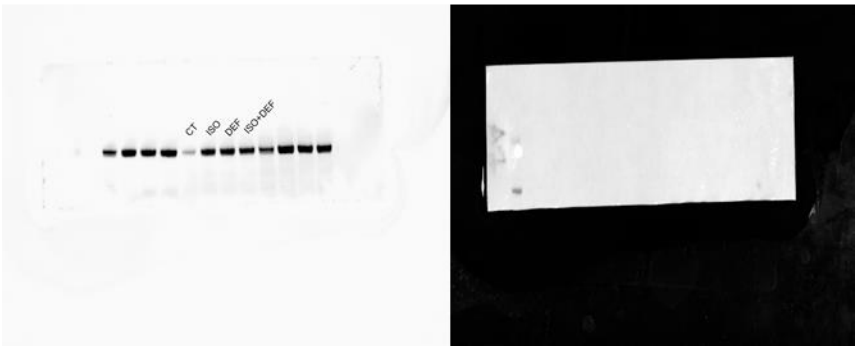

DMT1

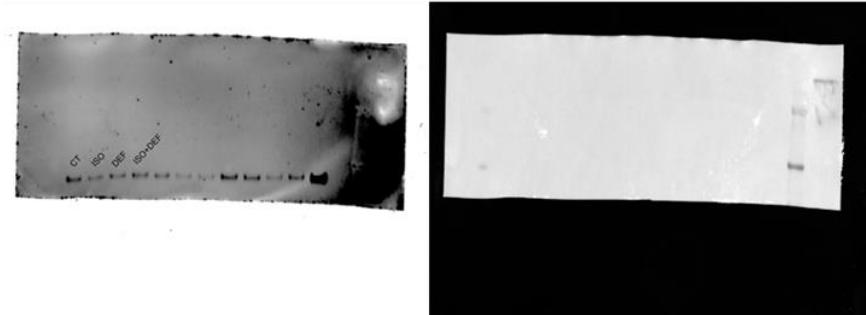

FPN

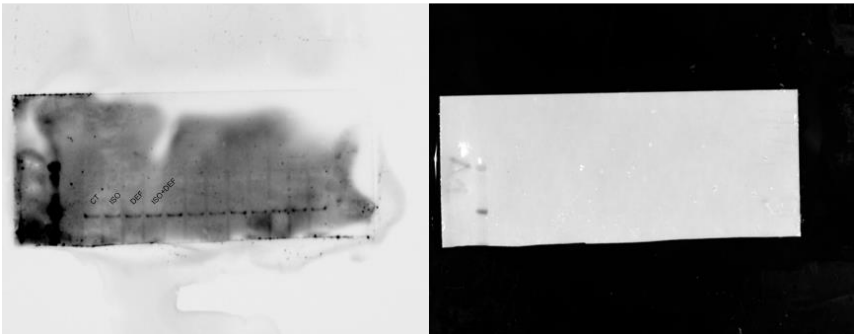

HEPCIDIN

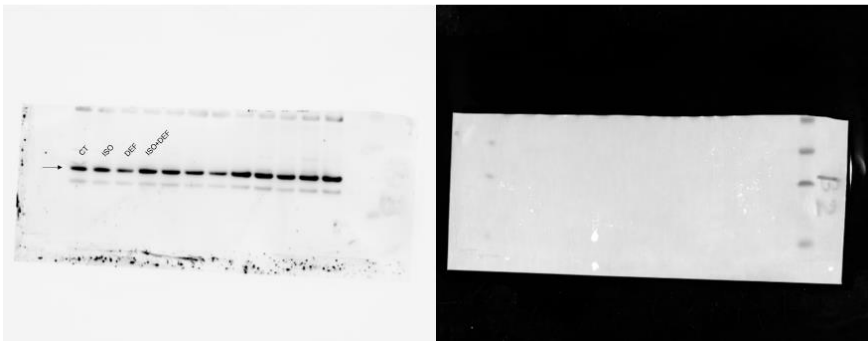

FTMT

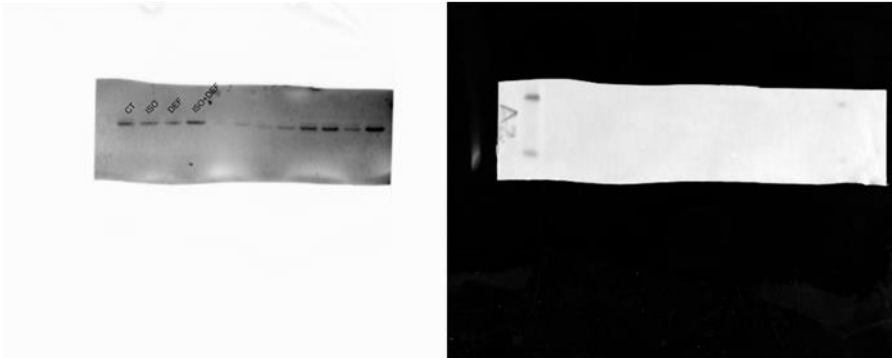

MFRN1

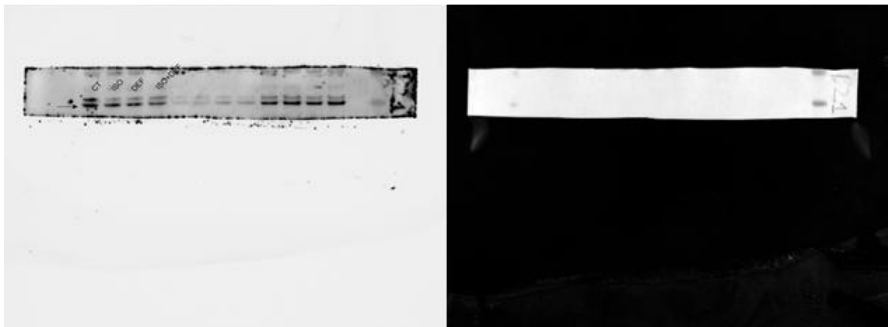

MFRN2

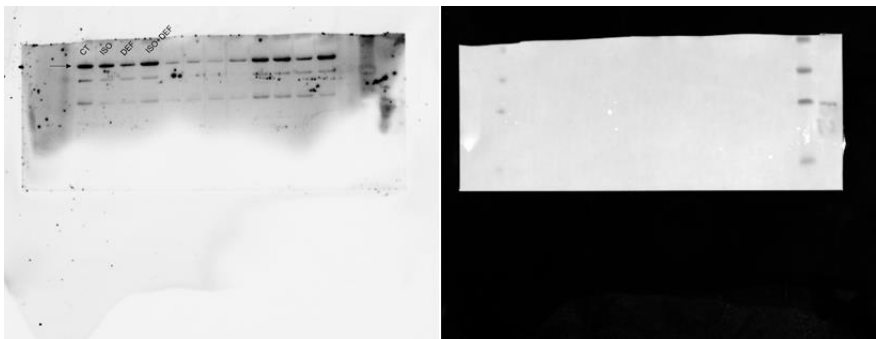

ABCB7

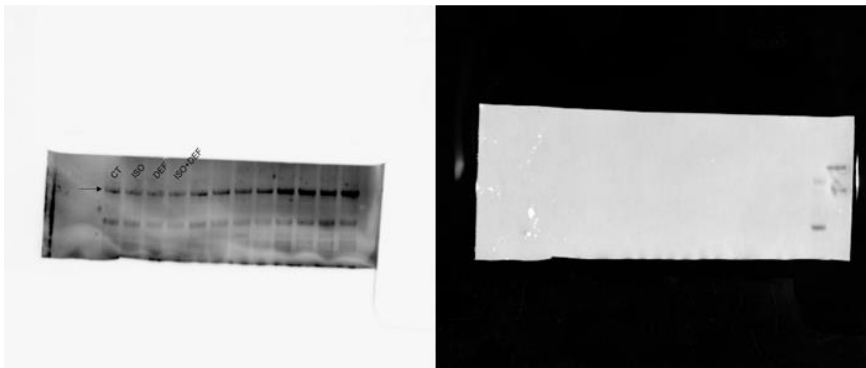

ABCB8

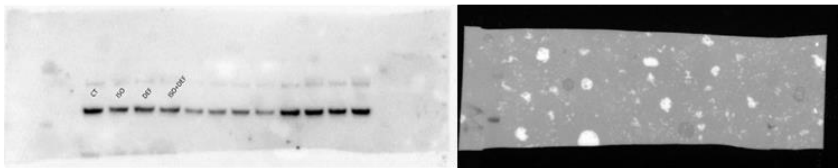

MFN1

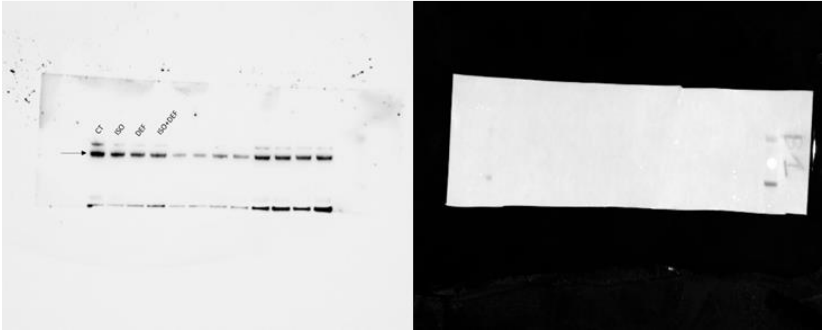

MFN2

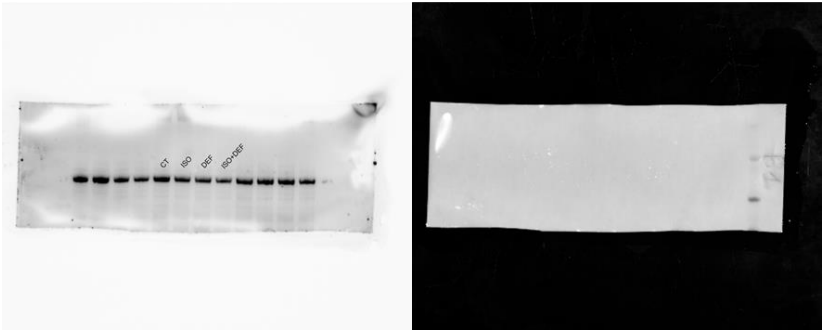

ACTIN

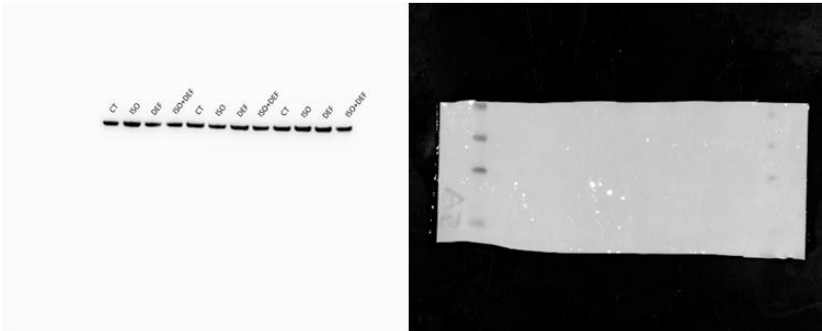

ACTIN

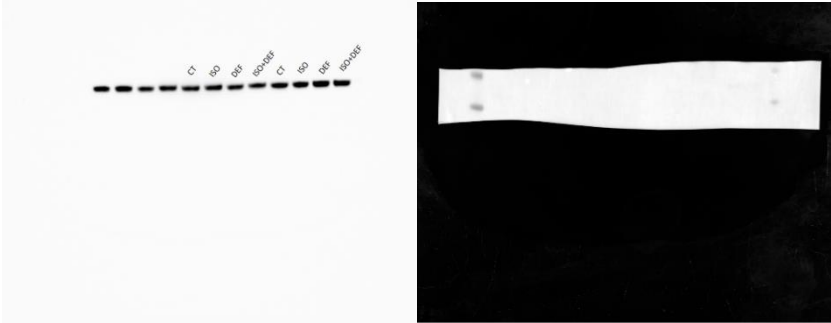

Supplement: Supplementary file 1 [file biomolecules-16-00582-s001.zip › biomolecules-4195121-File S1.pdf]
